# Concurrence of oral and genital human papillomavirus infection in healthy men: a population-based cross-sectional study in rural China

**DOI:** 10.1038/srep15637

**Published:** 2015-10-27

**Authors:** Fangfang Liu, Dong Hang, Qiuju Deng, Mengfei Liu, Longfu Xi, Zhonghu He, Chaoting Zhang, Min Sun, Ying Liu, Jingjing Li, Yaqi Pan, Tao Ning, Chuanhai Guo, Yongmei Liang, Ruiping Xu, Lixin Zhang, Hong Cai, Yang Ke

**Affiliations:** 1 Key Laboratory of Carcinogenesis and Translational Research (Ministry of Education/Beijing), Laboratory of Genetics, Peking University Cancer Hospital & Institute, Beijing, China; 2 Department of Epidemiology and Biostatistics, School of Public Health, Nanjing Medical University, Nanjing, China; 3 Department of Epidemiology, School of Public Health, University of Washington, Seattle, WA, USA; 4 Department of Pathology, School of Medicine, University of Washington, Seattle, WA, USA; 5 Anyang Cancer Hospital, Anyang, Henan Province, People’s Republic of China

## Abstract

Human papillomavirus (HPV) infection, a primary cause of genital cancer, is also related to the increasing incidence of oropharyngeal cancer among young men. Relatively little is known about the concurrence of oral and genital infection among healthy individuals. Oral and genital swab exfoliated cells were collected simultaneously from 2566 men in rural China. Using general primer-mediated (SPF1/GP6+) PCR and sequencing, HPV testing results were obtained from 2228 men with both valid oral and genital specimens (β-globin-positive). The prevalence of HPV infection was 6.7% in the oral cavity and 16.9% for the external genitalia. Among 43 men (1.9%, 43/2228) with oral-genital coinfection, 60.5% (26/43) harbored an identical HPV type at both sites. The risk of oral HPV infection was higher among men with genital infection than among uninfected men (11.4% *vs.* 5.7%, Adjusted OR = 2.3, 95% CI: 1.6–3.4). In addition, having multiple lifetime sexual partners was a significant risk for oral-genital HPV coinfection (Adjusted OR = 2.6, 95% CI: 1.0–7.0; 2 partners *vs.* 1 partner). These findings provide a basis for further understanding the natural history and transmission dynamics of oral HPV infection.

Human papillomavirus (HPV) infection is an etiologic factor for cervical, anal and penile cancer^1^. More recent evidence shows that HPV also causes a subset of head and neck cancer including 20–90% of oropharyngeal cancer^2^. In 2008, approximately 85,000 cases of oropharyngeal cancer were diagnosed globally, with more than 22,000 cases being HPV positive^2^. Notably, the incidence of HPV-related oropharyngeal cancer is significantly increasing, particularly in young men^3^,^4^. The prevalence of oral HPV infection in healthy individuals ranges from 1.3% to 9.2% worldwide, with HPV-16 being the most prevalent oncogenic type^5^. Previous studies have suggested that individuals with high-risk sexual behaviors (e.g. practicing oral sex and having multiple sexual partners) are at a greater risk for oral HPV infection^5^,^6^,^7^,^8^,^9^.

However, whether oral HPV infection is associated with genital infection remains largely unknown, especially among men. Previous studies investigating the association of oral and genital HPV infection among men were based largely on particular high-risk groups such as university students, sexually transmitted disease clinic patients, HIV-infected men, or homosexual men^10^,^11^,^12^,^13^,^14^,^15^,^16^. These studies, mostly limited in sample size, have indicated that concurrent oral-genital HPV infections are rare and have reported inconsistent results regarding type-specific association of HPV infection at these two sites^10^,^11^,^12^,^13^,^14^,^15^,^16^.

Concurrence of oral and genital HPV infection has rarely been investigated in a general male population. Previously, we reported an overall oral HPV prevalence of 6% in 5410 25–65 year old healthy males and females in rural China^17^. The intent of this study is to evaluate the association of HPV DNA simultaneously present in collected male external genital and oral specimens from this general population and identify relevant risk factors.

## Results

### Participant characteristics

Among 2721 eligible subjects, 2228 (81.9%) participants showed valid HPV DNA results for both oral and genital specimens. The 493 individuals not included in analysis were either non-responding (n = 155) or were negative for beta-globin (n = 338). These excluded subjects were more likely to work outside of the local area, show loss of teeth, have a history of oral disease, and report oral sex practices, but were less likely to have a smoking history (Supplementary Table S1). The median age of the participants was 43 years (range, 25–65 years), and half of these individuals were engaged in farming (45.3%) (Table 1). Most subjects were married or cohabiting (94.7%), and had a level of education of less than 9 years total (88.6%). Both cigarette smoking (65.9%) and alcohol consumption (36.4%) were common. The proportion of individuals who reported having lost >10 teeth or who reported a history of oral disease were 8.3% and 1.8% respectively. With respect to sexual behaviors, 81.7% reported occasionally or never washing external genitalia before sex, 14.6% reported having ≥2 lifetime sexual partners, and 3.9% had a history of oral sex practices.

### HPV detection and type-specific concordance

Among 2228 men, 376 (16.9%) and 149 (6.7%) were positive for genital and oral HPV infection, respectively (Table 1). Compared with oral infections, the spectrum of HPV types on the external genitalia (43 types) was more diverse and included all 11 HPV types which were detected in oral specimens. A predominance of non-oncogenic HPV types was observed both in the oral cavity and on the external genitalia. HPV-16 was the most prevalent type of oncogenic HPV (oral cavity: 0.45%; external genitalia: 2.83%), and HPV-3 was the most prevalent type of non-oncogenic HPV (oral cavity: 4.53%; external genitalia: 3.37%) (Supplementary Table S2).

The prevalence of oral HPV infection among men without genital HPV infection was 5.7%. Of the men with HPV detected on the genitalia, 11.4% also had an oral HPV infection. Among 43 men (1.9%, 43/2228) positive for HPV in both oral and genital specimens, 37.2% (16/43) had no types in common, 62.8% (27/43) were concordant for at least one type, and 60.5% (26/43) harbored exactly the same HPV type at both sites, including 18 men concordant for HPV-3, 4 men concordant for HPV-10, 3 men concordant for HPV-57, and 1 man concordant for HPV-29 (Table 2). The Monte-Carlo simulation showed that the observed events of positive concordance significantly exceeded the expected value (27 *vs.* 0.6, *P* < 0.001) (Supplementary Table S3).

### Association between oral and genital HPV infections

Overall, the prevalence of oral HPV infection was higher among men with genital infection than among those without genital HPV infection (11.4% *vs.* 5.7%, Adjusted OR = 2.3, 95% CI: 1.6–3.4) (Table 3). In the type-specific analysis, the presence of one specific HPV type in oral specimens was associated with the presence of the same type in genital specimens (Adjusted OR = 55.7, 95% CI: 36.1–86.0).

### Risk factor analysis

Men with multiple lifetime sexual partners were found to be at a higher risk for oral-genital HPV coinfection (Adjusted OR = 2.6, 95% CI: 1.0–7.0; 2 partners *vs.* 1 partner), as was the case for genital HPV infection (Table 1).

## Discussion

This population-based investigation of the concordance of HPV infections in the oral cavity and on the external genitalia is to our knowledge the first such investigation to be undertaken among Chinese men. Although oral-genital coinfection in healthy men was a rare event in this study (1.9%), over 60.0% of these concurrent infections were completely type-specific concordant, and oral HPV prevalence was significantly higher among men with genital HPV infection than among those without. These data provide a basis for further understanding the natural history and transmission dynamics of oral HPV infection.

Prevalence of oral HPV was significantly lower than on the external genitalia in healthy adult Chinese men, similar to what has been observed in other populations^6^. The distinct microenvironment of the oral cavity including the existence of the abundant antimicrobial agents in the saliva may in part explain the overall lower prevalence of infection and less diversity in oral HPV types^18^,^19^. Given the low prevalence of oral HPV infection (6.7%), it is not surprising that concurrent oral-genital HPV infection (1.9%) was uncommon in this study. Despite this rarity of coinfection, a relatively high level of type-specific concordance (62.8%, 27/43) was observed among concurrent oral and genital infections, in keeping with some previous studies^10^,^16^. It is of note that coinfection was only observed for non-oncogenic HPV in this population. The absence of concurrent infections with oncogenic HPV may be due to the lower frequency of oncogenic HPV infection in the oral cavity (0.5%), in agreement with the relatively low incidence of HPV-related oropharyngeal cancer^20^.

Among concurrent non-oncogenic HPV types found in this population, HPV-3, which has been rarely reported elsewhere, was the most dominant type. Since antibody against HPV-3 L1 was also frequently detected in serum from the same cohort^21^, it is possible that this type is relatively more prevalent in China. Apart from differences in populations, the frequent identification of HPV-3 may be partly explained by the use of different HPV testing methods. Compared to hybridization with pre-assigned probes, the method adopted in this study using general primer mediated PCR followed by sequencing may maximize identification of the spectrum of HPV types including HPV-3. As a member of Alpha-2 clade, HPV-3 is typically found in benign forms of epidermodysplasia verruciformis (EV) and can induce flat, wart-like lesions over the body^22^,^23^. However, the pathological consequences of HPV-3 infection in oral mucosa remain unknown and warrant further investigation.

In this study, significantly higher prevalence of type-specific HPV in oral specimens was found among men positive for genital HPV of the same type, which is consistent with some but not all previous surveys^10^,^14^,^16^. The biological mechanism of this correlation is unclear. However, it may reflect the fact that HPV infections at these two sites in the same individual interact with each other and thus share similarities in their natural history of infection. Oral sex with a genital HPV-positive partner, or oral-oral contact with an oral HPV-positive partner may give rise to concurrent infection with this observed type specificity. In addition to partner to partner transmission, it is also possible that HPV infection at one site can be acquired by autoinoculation from the other site (e.g. via fingers) in the same individual^11^. In support of the clinical significance of the association between oral and genital HPV infection, a study using the Surveillance, Epidemiology, and End Results (SEER) cancer database of United States found that while there was no elevated risk of HPV-unrelated cancer (e.g. prostate or bladder) following anogenital cancer, the incidence of a second primary oral cavity/pharyngeal cancer was significantly increased following an index genital cancer^24^. In order to develop more efficient and cost-effective strategies for control of HPV-associated cancer, further research is warranted to pinpoint the major route(s) of oral-genital HPV transmission.

Many studies have suggested that both oral and genital HPV infections are mainly sexually transmitted^7^,^25^. However, studies regarding the risk factors for concurrence of oral-genital HPV infections are few in number. As expected, we found that individuals with multiple lifetime sexual partners have a higher likelihood of coinfection. Nevertheless, we did not find a significant association of oral sex and oral-genital coinfection. This result is similar with that in our previous study of oral HPV infection^17^. The possibility of self-reporting bias which might lead to exposure misclassification and thus result in dilution of risk estimates to the null cannot be excluded. However, efforts were made to minimize this bias (e.g. conducting interviews in private settings). Another possible explanation is insufficient statistical power, as oral sex is rare among rural Chinese population whose sexual activity is considerably more conservative as compared with westerners. Recent studies have observed an absence of any relationship of oral sex and oral HPV infection^6^,^26^, implying there are alternative modes of transmission (nonsexual routes). Thus, more adequately-powered studies are needed to clarify these associations.

This study has limitations. First, the nonparticipation and exclusion of participants because of specimen inadequacy may undermine the validity for generalizability of study results to some extent. Second, self-reported sexual behavior features may be subject to reporting bias. Additionally, despite a large population sample size, the low prevalence of oral-genital coinfection may limit our ability to identify some associations. This low prevalence of coinfection may also have rendered subgroup analysis of carcinogenic HPV impossible. Finally, due to the cross-sectional nature of this study, temporal relationship cannot be inferred. A longitudinal cohort study with rigorous follow-up is needed to evaluate the relationship of HPV acquisition at different anatomic sites and its relevant factors.

In conclusion, our data suggested that HPV infections of the oral cavity and the male genitalia in the same individual were type-specifically correlated. Concurrence of oral-genital infections was associated with multiple sexual partners. Given that HPV-related oropharyngeal cancer appears to be on the increase, improved insight into the relationship of HPV infection at the oral cavity and other anatomical sites may help to inform and direct future prevention efforts, such as HPV vaccination.

## Materials and Methods

### Study population

A population-based prospective cohort study of esophageal cancer and its associated determinants has been published previously^27^. The current investigation was carried out in 6 of the 9 villages composing the parent cohort study in 2009–2011. Eligibility criteria were as follows: 1) male permanent residents in the target villages; 2) 25–65 years of age; 3) no prior diagnosis of cancer, mental illness, or cardiovascular disease; and 5) no history of HBV, HCV, or HIV infection. All participants provided written informed consent, and the study was approved by the Institutional Review Board of the School of Oncology, Peking University. The methods were carried out in accordance with the approved guidelines.

### Specimen and data collection

Exfoliated cells from oral cavity and genitalia were collected by an experienced clinician using saline-moistened cotton swabs, as described previously^17^,^25^. Oral specimens were collected from the buccal mucosa, palate, top and bottom of the tongue, the inner upper and lower lips, and the gingival surfaces by rubbing five times at each site. Exfoliated cells from the male genital shaft, glans penis, coronal sulcus, and scrotum were collected in the same manner. The cells collected on these swabs were then rinsed in 0.9% saline solution. After centrifugation at 3000 g for 10 minutes at 4 °C, the supernatant was decanted and the cell pellet was frozen at −70 °C until DNA was extracted.

A one-on-one computer-aided interview was carried out by a trained interviewer of male gender. Information regarding demographic characteristics, cigarette consumption (defined as 1 cigarette or more each day for at least 1 year), alcohol consumption (defined as use of Chinese liquor twice each week or more for at least 1 year), characteristics of sexual behavior (history of oral sex practices and lifetime number of sexual partners), and self-reported history of oral disease in the preceding 12 months (e.g. ulcers, gum disease, or chronic inflammation) was collected. Numbers of missing teeth were also recorded.

### Laboratory procedure

DNA was extracted and purified on a Biomek 3000 automated workstation with the E.Z.N.A.TM Mag-Bind Tissue DNA Kit (Omega Bio-Tek, Inc.). The β-globin gene was evaluated in all purified DNA samples (forward primer, 5′-GAAGAGCCAAGGACAGGTAC-3′; reverse primer, 5′-CAACTTCATCCAGGTTCACC-3′). β-globin PCR mixture contained 50 ng template DNA, 0.2 μM each primer, 0.2 mM deoxynucleoside triphosphate, 1 × PCR buffer containing 1.5 mM MgCl_2_, and 0.8 U HotStarTaq polymerase (Qiagen, Germany). After a 15-min enzyme activation at 95 °C, 40 cycles of amplication (94 °C for 40 s, 58 °C for 40 s, and 72 °C for 40 s) and a final extension at 72 °C for 10 min were carried out. Detection of HPV DNA was performed for the β-globin positive samples by amplifying a fragment of 184 bp in the L1 gene using a highly sensitive PCR primer set (SPF1/GP6+). The PCR was performed in a reaction mixture containing 50 ng template DNA, 0.4 μM each primer, 0.15 mM deoxynucleoside triphosphate, 1 × PCR buffer containing 1.5 mM MgCl_2_, and 0.8 U HotStarTaq polymerase. PCR conditions were as follows: preheating for 15 min at 95 °C, 45 cycles of amplification (94 °C for 40 s, 49 °C for 50 s, and 72 °C for 30 s), and a final extension at 72 °C for 10 min. PCR products of HPV positive specimens were purified and then directly sequenced using an ABI 3730XL DNA Analyzer^25^. A broad spectrum of HPV types was identifiable using SPF1/GP6+-mediated PCR and sequencing method (Oncogenic: HPV-16, 18, 26, 31, 35, 39, 45, 51, 52, 56, 58, 59, 66, and 68; non-oncogenic: HPV-2, 3, 6, 7, 10, 11, 27, 29, 30, 32, 33, 37, 40, 42, 43, 44, 53, 54, 55, 57, 61, 62, 67, 69, 70, 72, 73, 74, 75, 77, 81, 82, 83, 84, 87, 89, 90, 91, 94, and 127)^22^,^25^,^28^. Samples were considered to be HPV positive if any of these types were detected. Rigorous quality control procedures were implemented to avoid potential contamination as previously described^17^,^29^.

### Statistical analysis

Kappa value, percent agreement, and percent positive agreement were calculated to assess the concordance of HPV infection in paired oral and genital specimens from the same individuals. The McNemar’s test was used to evaluate the difference in the positivity of HPV infection in the oral cavity and on the external genitalia. To evaluate type-specific positive concordance, the analyzed observations were restricted to those positive for at least one site (excluding observations which were negative for HPV infection at both oral cavity and external genitalia) and a Monte-Carlo simulation method (1000 iterations) was used for comparisons of observed concordant events and expected value^30^,^31^.

To identify independent determinants for HPV infection, exposure variables that showed statistical significance in univariate logistic regression analysis, together with factors previously reported to be relevant (e.g., age, gender, smoking, and sexual behaviors), were included in multivariate logistic regression models (Variance inflation factor was used to examine whether variables in multivariate models were collinear). To examine linear trends, ordered categorical variables were treated as continuous covariates in the regression analysis.

For type-specific analysis to assess whether the presence of one specific HPV type in the oral cavity was associated with the presence of the same type in the genitalia, generalized estimating equations with an exchangeable correlation structure were used to account for possible correlations of different types of HPV. For groups of HPV types (i.e. any HPV type), infection of each type among the analyzed group for each individual was treated as one observation in the type-specific analysis. For instance, for any HPV type, each subject could have 43 HPV-type outcomes (a total of 43 HPV types were detected in this population).

All tests were two-sided and differences with *P* <0.05 were considered statistically significant. Statistical analyses were conducted using Stata 11.0 (Stata Corp.).

## Additional Information

**How to cite this article**: Liu, F. *et al.* Concurrence of oral and genital human papillomavirus infection in healthy men: a population-based cross-sectional study in rural China. *Sci. Rep.* **5**, 15637; doi: 10.1038/srep15637 (2015).

## Supplementary Material

Supplementary Table S1-S3

## Figures and Tables

**Table 1:** Prevalence of HPV infection by anatomic site and selected characteristics in men of rural Anyang, China, 2009–2011.

Variables	No. (%)	Oral cavity (N = 2228)			External genitalia (N = 2228)			Oral cavity and external genitalia (N = 43)^a^		
		%	Crude OR (95% CI)^b^	Adjusted OR (95% CI)^b^	%	Crude OR (95% CI)^b^	Adjusted OR (95% CI)^b^	%	Crude OR (95% CI)^b^	Adjusted OR (95% CI)^b^
Total	2228 (100.0)	6.7			16.9			1.9		
Age, years										
25–35	515 (23.1)	6.4	1.0	1.0	14.6	1.0	1.0	1.6	1.0	1.0
36–45	792 (35.6)	6.2	1.0 (0.6–1.5)	1.0 (0.6–1.6)	19.3	1.4 (1.0–1.9)	1.6 (1.2–2.2)	2.4	1.6 (0.7–3.6)	1.7 (0.7–4.0)
46–55	458 (20.6)	6.8	1.1 (0.6–1.8)	1.2 (0.7–2.1)	16.4	1.1 (0.8–1.6)	1.4 (0.9–2.0)	2.2	1.4 (0.6–3.6)	1.4 (0.5–3.9)
56–65	463 (20.8)	7.8	1.2 (0.8–2.0)	1.3 (0.7–2.3)	15.8	1.4 (0.8–1.6)	1.4 (0.9–2.1)	1.3	0.8 (0.3–2.4)	1.0 (0.3–3.5)
*P* value for trend^d^			0.340	0.363		0.972	0.251		0.686	0.963
Education level										
Illiteracy, <1 year	120 (5.4)	10.0	1.0	1.0	18.3	1.0	1.0	1.7	1.0	1.0
Primary school, 1–6 years	618 (27.7)	7.3	0.7 (0.4–1.4)	0.7 (0.3–1.4)	16.4	0.9 (0.5–1.5)	0.9 (0.5–1.5)	1.0	0.6 (0.1–2.9)	0.6 (0.1–3.2)
Junior high school, 7–9 years	1236 (55.5)	6.2	0.6 (0.3–1.1)	0.6 (0.3–1.2)	17.2	0.9 (0.6–1.5)	0.9 (0.5–1.6)	2.5	1.5 (0.4–6.4)	1.5 (0.3–7.3)
Senior high school or above, >9 years	254 (11.4)	6.4	0.6 (0.3–1.3)	0.6 (0.3–1.4)	15.9	0.8 (0.5–1.5)	0.8 (0.4–1.5)	1.6	1.0 (0.2–5.3)	0.9 (0.1–5.8)
*P* value for trend^d^			0.150	0.222		0.828	0.666		0.228	0.332
Marital status										
Married or cohabiting	2110 (94.7)	6.7	1.0	1.0	16.6	1.0	1.0	1.9	1.0	1.0
Not married, divorced, separated or widowed	118 (5.3)	5.9	0.9 (0.4–1.9)	0.7 (0.3–1.6)	22.0	1.4 (0.9–2.2)	1.5 (1.0–2.4)	2.5	1.4 (0.4–4.4)	1.6 (0.5–5.5)
Type of employment										
Farming at home	1010 (45.3)	6.3	1.0	1.0	15.7	1.0	1.0	1.9	1.0	1.0
Working in local area	366 (16.4)	6.3	1.0 (0.6–1.6)	1.1 (0.6–1.8)	16.7	1.1 (0.8–1.5)	1.0 (0.7–1.5)	1.4	0.7 (0.3–1.9)	0.7 (0.2–1.8)
Working outside local area	662 (29.7)	7.1	1.1 (0.8–1.7)	1.2 (0.8–1.9)	16.9	1.1 (0.8–1.4)	1.0 (0.8–1.4)	2.1	1.1 (0.6–2.3)	1.0 (0.5–2.1)
Other	190 (8.5)	7.9	1.3 (0.7–2.3)	1.5 (0.8–2.8)	23.2	1.6 (1.1–2.4)	1.5 (1.0–2.2)	2.6	1.4 (0.5–3.8)	1.2 (0.4–3.5)
Cigarette smoking^c^										
Never	760 (34.1)	6.8	1.0	1.0	14.6	1.0	1.0	2.0	1.0	1.0
Ever	1468 (65.9)	6.6	1.0 (0.7–1.4)	0.9 (0.7–1.4)	18.1	1.3 (1.0–1.6)	1.3 (1.0–1.6)	1.9	1.0 (0.5–1.8)	1.0 (0.5–2.0)
Alcohol consumption^c^										
Never	1418 (63.6)	7.1	1.0	1.0	15.7	1.0	1.0	1.9	1.0	1.0
Ever	810 (36.4)	6.1	0.8 (0.6–1.2)	0.8 (0.6–1.2)	19.0	1.3 (1.0–1.6)	1.1 (0.9–1.4)	2.0	1.0 (0.6–1.9)	1.0 (0.5–1.8)
History of oral disease^c^										
Never	2188 (98.2)	6.5	1.0	1.0	17.0	—	—	2.0	—	—
Ever	40 (1.8)	15.0	2.5 (1.0–6.1)	2.8 (1.1–7.1)	10.0	—	—	0.0	—	—
Number of missing teeth										
None	382 (17.2)	7.1	1.0	1.0	18.0	—	—	2.1	1.0	1.0
1–10	1639 (73.6)	6.5	0.9 (0.6–1.4)	0.9 (0.6–1.4)	16.6	—	—	1.9	0.9 (0.4–2.0)	1.0 (0.4–2.1)
>10	185 (8.3)	7.0	1.0 (0.5–2.0)	0.8 (0.4–1.7)	15.7	—	—	2.2	1.0 (0.3–3.5)	1.6 (0.4–6.2)
*P* value for trend^d^			0.890	0.551		—	—		0.965	0.694
Wash external genitalia before sex										
Occasionally or never	1820 (81.7)	6.9	1.0	1.0	16.5	1.0	1.0	1.9	1.0	1.0
Often or every time	408 (18.3)	5.6	0.8 (0.5–1.3)	0.9 (0.5–1.4)	18.6	1.2 (0.9–1.5)	1.2 (0.9–1.6)	2.2	1.2 (0.6–2.5)	1.2 (0.5–2.5)
Oral sex practices										
Never	2141 (96.1)	6.5	1.0	1.0	16.9	1.0	1.0	2.0	1.0	1.0
Ever	87 (3.9)	10.3	1.6 (0.8–3.4)	1.7 (0.8–3.5)	16.1	0.9 (0.5–1.7)	0.8 (0.4–1.5)	1.1	0.6 (0.1–4.3)	0.6 (0.1–4.9)
Lifetime number of sexual partners										
0–1	1903 (85.4)	6.7	1.0	1.0	14.8	1.0	1.0	1.7	1.0	1.0
2	115 (5.2)	7.0	1.0 (0.5–2.2)	1.1 (0.5–2.3)	25.2	1.9 (1.2–3.0)	1.9 (1.2–3.0)	4.3	2.6 (1.0–6.7)	2.6 (1.0–7.0)
≥3	210 (9.4)	6.7	1.0 (0.6–1.8)	0.9 (0.5–1.7)	31.0	2.6 (1.9–3.5)	2.7 (1.9–3.7)	2.4	1.4 (0.5–3.6)	1.5 (0.6–4.0)
*P* value for trend^d^			0.975	0.918		<0.001	<0.001		0.241	0.197

Abbreviation: HPV, human papillomavirus; OR, odds ratio; CI, confidence interval.

“—” denotes that the indicated variables were not included in the logistic regression models.

^a^Data for the oral cavity and external genitalia category include the subset of men with HPV in both sites.

^b^Adjusted ORs and 95% CIs were calculated by multivariate logistic regression models including all listed variables.

^c^Cigarette smoking was defined as consuming an average of one cigarette or more per day for ≥12 months, and alcohol consumption was defined as drinking Chinese liquor at least twice per week for ≥12 months. A history of oral disease was defined as self-reported oral ulcers, gum disease, or chronic oral inflammation in the preceding 12 months.

^d^*P* values for trends were derived by logistic regression analyses taking categorical variables as continuous variables.

**Table 2:** Concordance and discordance of type-specific HPV types detected in paired oral and genital specimens from individual male participants in rural Anyang, China, 2009–2011.

HPV status	No. (%)	Genital HPV types	Oral HPV types
Genital−/Oral−a	1746 (78.4)	None	None
Genital+/Oral−a	333 (15.0)	Types not shown	Types not shown
Genital−/Oral+^a^	106 (4.8)	Types not shown	Types not shown
Genital+/Oral+^a^	43 (1.9)		
Full concordance	26 (1.1)		
	18	HPV-3	HPV-3
	4	HPV-10	HPV-10
	3	HPV-57	HPV-57
	1	HPV-29	HPV-29
Partial concordance	1 (0.0)	HPV-3 and 16	HPV-3
No concordance	16 (0.7)		
	4	HPV-16	HPV-3
	1	HPV-18	HPV-3
	1	HPV-33	HPV-3
	1	HPV-43	HPV-3
	1	HPV 84	HPV-3
	1	HPV-91	HPV-3
	1	HPV-30	HPV-10
	1	HPV-33	HPV-16
	1	HPV-3	HPV-75
	1	HPV-18	HPV-75
	1	HPV-66	HPV-75
	1	HPV-16 and 73	HPV-3
	1	HPV-43 and 45	HPV-3

Abbreviation: HPV, human papillomavirus.

^a^Kappa = −1.460; Percent agreement = 80.3%; Percent positive agreement = 8.9%; McNemar chi-square = 117.380, *P* < 0.001; Pearson chi-square = 16.345, *P* < 0.001.

**Table 3:** Association of HPV infection in paired oral and genital specimens from individual male participants in rural Anyang, 2009–2011.

Analysis	HPV status of external genitalia	HPV status of oral cavity		Total	Crude OR (95% CI)^a^	Adjusted OR (95% CI)^b^
		Negative	Positive			
Overall						
	Negative	1746	106	1852	1.0	1.0
	Positive	333	43	376	2.1 (1.5, 3.1)	2.3 (1.6–3.4)
	Total	2079	149	2228		
Type-specific^c^						
	Negative	95247	405	95652	1.0	1.0
	Positive	125	27	152	50.8 (33.1, 77.8)	55.7 (36.1, 86.0)
	Total	95372	432	95804		

Abbreviation: HPV, human papillomavirus; OR, odds ratio; CI, confidence interval.

^a^Crude ORs and 95% CIs were calculated by univariate logistic regression analyses with generalized estimating equations (GEE).

^b^Adjusted ORs and 95% CIs were calculated by multivariate logistic regression analyses with GEE including age, education level, marital status, type of employment, cigarette smoking, alcohol consumption, history of oral disease, number of missing teeth, frequency of washing genitalia before sex, oral sex practices, and lifetime number of sexual partners.

^c^Number of observations for type-specific analysis = 43 (total number of types detected among oral and genital specimens) × 2228 (number of individuals).
