# Supplementary material for: Concurrence of oral and genital human papillomavirus infection in healthy men: a population-based cross-sectional study in rural China
Source: Sci Rep. 2015 Oct 27;5:15637. doi: 10.1038/srep15637 (PMC4621523; doi:10.1038/srep15637)
Supplement: Supplementary Table S1-S3 [file srep15637-s1.doc]

**Concurrence of oral and genital human papillomavirus infection in healthy men: a population-based cross-sectional study in rural China**

Fangfang Liu, Dong Hang, Qiuju Deng, Mengfei Liu, Longfu Xi, Zhonghu He, Chaoting Zhang, Min Sun, Ying Liu, Jingjing Li, Yaqi Pan, Tao Ning, Chuanhai Guo, Yongmei Liang, Ruiping Xu, Lixin Zhang, Hong Cai and Yang Ke

| **Supplementary Table S1. Distribution of socio-demographic characteristics of the male subjects included and not included in analysis in rural Anyang, China, 2009-2011** | | | | |
| --- | --- | --- | --- | --- |
| Variables | | Included in analysis^a^ (N=2228) | Not included in analysis^b^ (N=493) | *P* value*^c^* |
|  |  | No. (%) | No. (%) |  |
| Age, years | |  |  |  |
|  | 25-35 | 515 (23.1) | 97 (19.7) | 0.393 |
|  | 36-45 | 792 (35.6) | 178 (36.1) |  |
|  | 46-55 | 458 (20.6) | 107 (21.7) |  |
|  | 56-65 | 463 (20.8) | 111 (22.5) |  |
| Education level | |  |  |  |
|  | Illiteracy, <1 year | 120 (5.4) | 24 (4.9) | 0.363 |
|  | Primary school, 1-6 years | 618 (27.7) | 113 (22.9) |  |
|  | Junior high school, 7-9 years | 1236 (55.5) | 280 (56.8) |  |
|  | Senior high school or above, >9 years | 254 (11.4) | 55 (11.2) |  |
|  | Unknown^d^ | 0 (0.0) | 21 (4.3) |  |
| Marital status | |  |  |  |
|  | Married or cohabiting | 2110 (94.7) | 445 (90.3) | 0.474 |
|  | Never married, divorced, separated or widowed | 118 (5.3) | 29 (5.9) |  |
|  | Unknown^d^ | 0 (0.0) | 19 (3.9) |  |
| Type of employment | |  |  |  |
|  | Farming at home | 1010 (45.3) | 189 (38.3) | <0.001 |
|  | Working in local area | 366 (16.4) | 97 (19.7) |  |
|  | Working outside local area | 662 (29.7) | 167 (33.9) |  |
|  | Other | 190 (8.5) | 21 (4.3) |  |
|  | Unknown^d^ | 0 (0.0) | 19 (3.9) |  |
| Cigarette smoking^e^ | |  |  |  |
|  | Never | 760 (34.1) | 184 (37.3) | 0.044 |
|  | Ever | 1468 (65.9) | 288 (58.4) |  |
|  | Unknown^d^ | 0 (0.0) | 21 (4.3) |  |
| Alcohol consumption^e^ | |  |  |  |
|  | Never | 1418 (63.6) | 294 (59.6) | 0.578 |
|  | Ever | 810 (36.4) | 178 (36.1) |  |
|  | Unknown^d^ | 0 (0.0) | 21 (4.3) |  |
| Number of missing teeth | |  |  |  |
|  | None | 382 (17.2) | 49 (9.9) | <0.001 |
|  | 1-10 | 1639 (73.6) | 388 (78.7) |  |
|  | >10 | 185 (8.3) | 53 (10.8) |  |
|  | Unknown^d^ | 22 (1.0) | 3 (0.6) |  |
| History of oral disease^e^ | |  |  |  |
|  | Never | 2188 (98.2) | 447 (90.7) | <0.001 |
|  | Ever | 40 (1.8) | 46 (9.3) |  |
| Wash external genitalia before sex | |  |  |  |
|  | Occasionally or never | 1820 (81.7) | 385 (78.1) | 0.065 |
|  | Often or every time | 408 (18.3) | 108 (21.9) |  |
| Oral sex practices | |  |  |  |
|  | Never | 2141 (96.1) | 436 (88.4) | <0.001 |
|  | Ever | 87 (3.9) | 36 (7.3) |  |
|  | Unknown^d^ | 0 (0.0) | 21 (4.3) |  |
| Lifetime number of sexual partners | |  |  |  |
|  | 0-1 | 1903 (85.4) | 418 (84.8) | 0.068 |
|  | 2 | 115 (5.2) | 15 (3.0) |  |
|  | ≥3 | 210 (9.4) | 35 (7.1) |  |
|  | Unknown^d^ | 0 (0.0) | 25 (5.1) |  |
| Total | | 2228 (100.0) | 493 (100.0) |  |
| ^a^ Subjects with available human papillomavirus infection data for both oral cavity and external genitalia were included in analysis. | | | | |
| ^b^ Subjects without human papillomavirus infection data either in oral cavity or on the external genitalia (including 155 non-responding population and 338 responding population negative for beta-globin) were not included in analysis. | | | | |
| ^c^ *P* values were calculated by comparing subjects included in analysis and those not included using the χ^2^ test. | | | | |
| ^d^ Unknown was not included in the χ^2^ test. | | | | |
| ^e^ Cigarette smoking was defined as consuming an average of one cigarette or more per day for ≥12 months, and alcohol consumption was defined as drinking Chinese liquor at least twice per week for ≥ 12 months. A history of oral disease was self-reported oral ulcers, gum disease, or chronic oral inflammation in the preceding 12 months. | | | | |

| **Supplementary Table S2. Prevalence of HPV infections on the external genitalia and in the oral cavity in men of rural Anyang, China, 2009-2011** | | |
| --- | --- | --- |
| HPV type | External genitalia (N=2228) | Oral cavity (N=2228) |
|  | No. of HPV infection (%) | No. of HPV infection (%) |
| Total | 376 (16.90) | 149 (6.70) |
| Oncogenic type^a^ | 148 (6.64) | 12 (0.54) |
| HPV16 | 63 (2.83) | 10 (0.45) |
| HPV18 | 27 (1.21) | — |
| HPV33 | 8 (0.36) | — |
| HPV35 | 4 (0.18) | — |
| HPV39 | 1 (0.04) | — |
| HPV45 | 11 (0.49) | 3 (0.13) |
| HPV52 | 6 (0.27) | — |
| HPV56 | 3 (0.13) | — |
| HPV58 | 18 (0.81) | — |
| HPV59 | 5 (0.22) | — |
| HPV66 | 3 (0.13) | — |
| HPV68 | 6 (0.27) | — |
| Non-oncogenic type^a^ | 260 (11.67) | 137 (6.15) |
| HPV2 | 1 (0.04) | — |
| HPV3 | 75 (3.37) | 101 (4.53) |
| HPV6 | 8 (0.36) | 1 (0.04) |
| HPV7 | 2 (0.09) | — |
| HPV10 | 12 (0.54) | 15 (0.67) |
| HPV11 | 3 (0.13) | 1 (0.04) |
| HPV27 | 2 (0.09) | — |
| HPV29 | 3 (0.13) | 2 (0.09) |
| HPV30 | 4 (0.18) | — |
| HPV32 | 4 (0.18) | — |
| HPV40 | 6 (0.27) | — |
| HPV42 | 4 (0.18) | — |
| HPV43 | 10 (0.45) | — |
| HPV54 | 26 (1.17) | — |
| HPV57 | 17 (0.76) | 10 (0.45) |
| HPV67 | 7 (0.31) | 1 (0.04) |
| HPV69 | 1 (0.04) | — |
| HPV70 | 1 (0.04) | — |
| HPV72 | 1 (0.04) | — |
| HPV73 | 1 (0.04) | — |
| HPV74 | 4 (0.18) | — |
| HPV75 | 2 (0.09) | 4 (0.18) |
| HPV81 | 20 (0.90) | — |
| HPV82 | 1 (0.04) | — |
| HPV83 | 1 (0.04) | — |
| HPV84 | 6 (0.27) | — |
| HPV87 | 11 (0.49） | — |
| HPV89 | 1 (0.04) | — |
| HPV90 | 28 (1.26) | — |
| HPV91 | 9 (0.40) | — |
| HPV94 | 6 (0.27) | 4 (0.18) |
| “—” denotes no indicated type-specific HPV infection detected in the indicated sites. | | |
| ^a^ Oncogenic and non-oncogenic types were classified based on their carcinogenicity in cervical cancer (18). | | |

| **Supplementary Table S3. Positive type-specific concordance of HPV infection in paired oral and genital specimens from individual male participants in rural Anyang, 2009-2011**^a^ | | | | | | |
| --- | --- | --- | --- | --- | --- | --- |
| HPV status of external genitalia | HPV status of oral cavity | | Total | Number of observed positive concordant events and 95% CI^b^ | Number of expected positive concordant events^c^ and 95% CI^b^ | *P*-value^c^ |
|  | Negative | Positive |  |  |  |  |
| Negative | — | 405 | 405 | 27.00 (1.77, 37.26) | 0.60 (0.55, 0.65) | <0.001 |
| Positive | 125 | 27 | 152 |  |  |  |
| Total | 125 | 432 | 557 |  |  |  |
| **Abbreviation:** HPV, human papillomavirus; CI, confidence interval; “—” denotes that the corresponding data was not included in the analysis of positive type-specific concordance. | | | | | | |
| ^a^ Infection of each type for each individual was treated as one observation in the type-specific analysis. Number of total observations for type-specific analysis (95804) = number of types detected among oral and genital specimens (43) × number of individuals (2228). Number of observations for type-specific analysis of positive concordance (557) = number of total observations (95804) - number of observations negative at both oral cavity and external genitalia (95247). | | | | | | |
| ^b^ 95% CI were estimated using a null linear regression model implemented with the Generalized Estimating Equation (GEE) with a robust sandwich estimator of covariance to adjust for repeat measurements. | | | | | | |
| ^c^ A Monte-Carlo simulation method (1000 iterations) was used for comparison of observed events and expected values. | | | | | | |
